# 5-(4-Fluoro­phen­yl)-2-furylmethyl *N*-(2,6-difluoro­benzo­yl)carbamate

**DOI:** 10.1107/S1600536808001359

**Published:** 2008-01-18

**Authors:** Ying Li, Yong-Qiang Ma, Zi-Ning Cui, Xin-Ling Yang, Yun Ling

**Affiliations:** a College of Science, China Agricultural University, Beijing, 100094, People’s Republic of China

## Abstract

The title compound, C_19_H_12_F_3_NO_4_, was synthesized by the reaction of 5-(4-fluoro­phen­yl)-2-furan­methanol and 2,6-difluoro­benzoyl­isocyanate. The seven atoms of the fluorophenyl group are disordered over two positions with site occupancy factors *ca* 0.6 and 0.4. The dihedral angle between the furan and fluorophenyl rings is 1.58°. In the crystal structure, the mol­ecules are linked *via *inter­molecular N—H⋯O hydrogen bonds to form chains.

## Related literature

For related literature, see: Grosscurt & Tipker (1980 ▶); Grugier *et al.* (2000 ▶); Li *et al.* (2007 ▶); Yang *et al.* (1997 ▶, 1998 ▶, 2002 ▶).
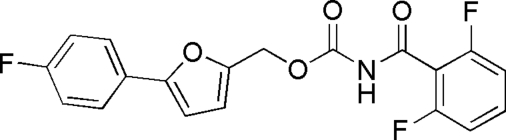


## Experimental

### 

#### Crystal data

C_19_H_12_F_3_NO_4_
*M* *_r_* = 375.30
Monoclinic, 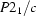

*a* = 7.5594 (11) Å
*b* = 12.9878 (19) Å
*c* = 17.332 (2) Å
β = 94.662 (2)°
*V* = 1696.0 (4) Å^3^
*Z* = 4
Mo *K*α radiation
μ = 0.13 mm^−1^
*T* = 294 (2) K
0.26 × 0.20 × 0.14 mm

#### Data collection

Bruker SMART CCD area-detector diffractometer
Absorption correction: multi-scan (*SADABS*; Sheldrick, 1996 ▶) *T*_min_ = 0.968, *T*_max_ = 0.983
9559 measured reflections
3453 independent reflections
2305 reflections with *I* > 2σ(*I*)
*R*_int_ = 0.026

#### Refinement

*R*[*F* ^2^ > 2σ(*F* ^2^)] = 0.038
*wR*(*F* ^2^) = 0.103
*S* = 1.00
3453 reflections
288 parameters
99 restraints
H-atom parameters constrained
Δρ_max_ = 0.16 e Å^−3^
Δρ_min_ = −0.17 e Å^−3^

### 

Data collection: *SMART* (Bruker, 1999 ▶); cell refinement: *SAINT* (Bruker, 1999 ▶); data reduction: *SAINT*; program(s) used to solve structure: *SHELXS97* (Sheldrick, 2008 ▶); program(s) used to refine structure: *SHELXL97* (Sheldrick, 2008 ▶); molecular graphics: *SHELXTL* (Sheldrick, 2008 ▶); software used to prepare material for publication: *SHELXTL*.

## Supplementary Material

Crystal structure: contains datablocks I, global. DOI: 10.1107/S1600536808001359/sg2221sup1.cif

Structure factors: contains datablocks I. DOI: 10.1107/S1600536808001359/sg2221Isup2.hkl

Additional supplementary materials:  crystallographic information; 3D view; checkCIF report

## Figures and Tables

**Table 1:** Hydrogen-bond geometry (Å, °)

*D*—H⋯*A*	*D*—H	H⋯*A*	*D*⋯*A*	*D*—H⋯*A*
N1—H1*A*⋯O3^i^	0.810 (17)	2.126 (18)	2.9129 (19)	164.0 (17)

Symmetry code: (i) 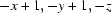.

**Fig. 1.**
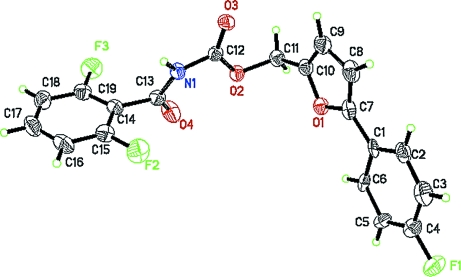
The molecular structure of (I), showing 40% probability displacement ellipsoids and the atom-numbering scheme.

**Fig. 2.**
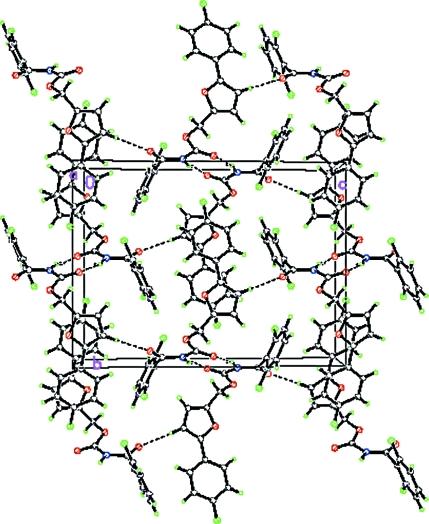
The crystal packing of (I). Intermolecular hydrogen bonds are shown as dashed lines.

## supplementary crystallographic information

### Comment

Chitin synthesis inhibitors, mainly included benzoylphenylureas and peptidyl nucleosides (Grugier *et al.*, 2000), have been widely used in agriculture and medicine owing to their excellent selectivity (Li *et al.*, 2007). Benzoylphenylureas, discovered in the 1970 s (Grosscurt & Tipker, 1980), are well known as insecticides, but only a few of them show antifungal activity. In order to find new fungicidal chemicals, based on our previous work (Yang *et al.*, 1997; Yang *et al.*, 1998; Yang *et al.*, 2002), 2,6- difluorobenzoyl carbamic acid-5-(4- fluorophenyl)-2-furanmethylester (I), and its analogues were designed through the modifications on the urea linkage of benzoylphenylureas. The compound (I) was synthesized by the reaction of 5- (4-fluorophenyl)-2-furanmethanol and 2,6-difluorobenzoylisocyanate. Finally in the preliminary bioassay, we found it showed obvious antifungal activity against different kinds of strains. To get more information about the structure and the mode of action, we prepared a single-crystal of (I) and its crystal will be reported herein. (I)

The molecular structure of the title compound is given in Fig.1. Single crystals showed clearly that some sort of disorder was present in the structure, containing the atoms C1, C2, C3, C4, C5, C6 and F1. The phenyl group was disordered in two positions with occupy factors 0.42 (3)/0.58 (3). The disordered phenyl group was constrained as a hexagon with C—C distances of 1.39 Å. This compound contains three ring planes: (*a*) composed of C14, C15, C16, C17, C18, C19, (*b*) composed of C7, C8, C9, C10, O1 and (*c*) composed of C1, C2, C3, C4, C5, C6. The dihedral angle between (*b*) and (*c*) is 1.58° which infers that the furan ring is almost coplanar with the adjacent benzene ring. In the crystal structure, the carboxyl O and amide NH are involved in N—H···O intermolecular hydrogen bonds. The molecules are linked *via* intermolecular N—H···O hydrogen bonds to form chains. The data is shown in Table1 and Fig.2.

### Experimental

To a solution of 5- (4-fluorophenyl)-2-furanmethanol (2.0 g, 10.4 mmol) dissolved in anhydrous toluene(20 ml), 2,6-difluorobenzoyl isocyanate (2.47 g, 13.5 mmol) was added. Then the mixture was stirred for 30 minutes at room temperature. Liquid was filtered off and the solid was dried. The solid was recrystallized from the solvent of petroleum ether and ethyl acetate (V _petroleumether_: V _ethyl acetate_ = 2.5: 1) and 2.87 g compound (I) was obtained in 73.5% yield. The colorless crystal was finally got after the second recrystallization.

### Refinement

H atoms were placed in calculated positions, with C—H = 0.93, 0.97 Å,and included in the final cycle of refinement using a riding model, with *U*_iso _(H) = 1.2*U*_eq_ (parent atom).

### Crystal data

**Table:**

C_19_H_12_F_3_NO_4_	*F*_000_ = 768
*M**_r_* = 375.30	*D*_x_ = 1.470 Mg m^−^^3^
Monoclinic, *P*2(1)/*c*	Mo *K*α radiation λ = 0.71073 Å
*a* = 7.5594 (11) Å	Cell parameters from 3023 reflections
*b* = 12.9878 (19) Å	θ = 2.8–23.5º
*c* = 17.332 (2) Å	µ = 0.13 mm^−^^1^
β = 94.662 (2)º	*T* = 294 (2) K
*V* = 1696.0 (4) Å^3^	Block, colourless
*Z* = 4	0.26 × 0.20 × 0.14 mm

### Data collection

**Table:**

Bruker SMART CCD area-detector diffractometer	3453 independent reflections
Radiation source: fine-focus sealed tube	2305 reflections with *I* > 2σ(*I*)
Monochromator: graphite	*R*_int_ = 0.026
*T* = 294(2) K	θ_max_ = 26.4º
phi and ω scans	θ_min_ = 2.0º
Absorption correction: multi-scan(SADABS; Sheldrick, 1996)	*h* = −9→6
*T*_min_ = 0.968, *T*_max_ = 0.983	*k* = −15→16
9559 measured reflections	*l* = −21→21

### Refinement

**Table:**

Refinement on *F*^2^	Secondary atom site location: difference Fourier map
Least-squares matrix: full	Hydrogen site location: inferred from neighbouring sites
*R*[*F*^2^ > 2σ(*F*^2^)] = 0.038	H-atom parameters constrained
*wR*(*F*^2^) = 0.103	*w* = 1/[σ^2^(*F*_o_^2^) + (0.0473*P*)^2^ + 0.2734*P*] where *P* = (*F*_o_^2^ + 2*F*_c_^2^)/3
*S* = 1.00	(Δ/σ)_max_ = 0.005
3453 reflections	Δρ_max_ = 0.16 e Å^−^^3^
288 parameters	Δρ_min_ = −0.17 e Å^−^^3^
99 restraints	Extinction correction: none
Primary atom site location: structure-invariant direct methods	

### Special details

**Table:**

Geometry. All e.s.d.'s (except the e.s.d. in the dihedral angle between two l.s. planes) are estimated using the full covariance matrix. The cell e.s.d.'s are taken into account individually in the estimation of e.s.d.'s in distances, angles and torsion angles; correlations between e.s.d.'s in cell parameters are only used when they are defined by crystal symmetry. An approximate (isotropic) treatment of cell e.s.d.'s is used for estimating e.s.d.'s involving l.s. planes.
Refinement. Refinement of *F*^2^ against ALL reflections. The weighted *R*-factor *wR* and goodness of fit *S* are based on *F*^2^, conventional *R*-factors *R* are based on *F*, with *F* set to zero for negative *F*^2^. The threshold expression of *F*^2^ > σ(*F*^2^) is used only for calculating *R*-factors(gt) *etc*. and is not relevant to the choice of reflections for refinement. *R*-factors based on *F*^2^ are statistically about twice as large as those based on *F*, and *R*- factors based on ALL data will be even larger.

### Fractional atomic coordinates and isotropic or equivalent isotropic displacement parameters (Å^2^)

**Table:**

	*x*	*y*	*z*	*U*_iso_*/*U*_eq_	Occ. (<1)
F1	0.413 (2)	−0.3113 (6)	0.0264 (9)	0.080 (2)	0.42 (3)
C1	0.2408 (19)	−0.0169 (4)	−0.0205 (6)	0.036 (2)	0.42 (3)
C2	0.309 (2)	−0.0746 (6)	−0.0790 (6)	0.056 (2)	0.42 (3)
H2A	0.3101	−0.0472	−0.1285	0.068*	0.42 (3)
C3	0.374 (2)	−0.1731 (6)	−0.0634 (7)	0.069 (3)	0.42 (3)
H3A	0.4192	−0.2116	−0.1025	0.082*	0.42 (3)
C4	0.3715 (17)	−0.2139 (5)	0.0107 (8)	0.054 (2)	0.42 (3)
C5	0.3038 (15)	−0.1563 (6)	0.0691 (6)	0.048 (2)	0.42 (3)
H5A	0.3023	−0.1836	0.1186	0.058*	0.42 (3)
C6	0.2385 (16)	−0.0578 (6)	0.0535 (5)	0.043 (2)	0.42 (3)
H6A	0.1932	−0.0192	0.0926	0.051*	0.42 (3)
F1'	0.4021 (18)	−0.3089 (5)	0.0520 (10)	0.109 (3)	0.58 (3)
C1'	0.2758 (17)	−0.0108 (4)	−0.0136 (5)	0.046 (2)	0.58 (3)
C2'	0.3377 (16)	−0.0798 (4)	−0.0665 (5)	0.0563 (19)	0.58 (3)
H2'A	0.3535	−0.0585	−0.1167	0.068*	0.58 (3)
C3'	0.3759 (14)	−0.1808 (4)	−0.0444 (7)	0.065 (2)	0.58 (3)
H3'A	0.4173	−0.2270	−0.0797	0.078*	0.58 (3)
C4'	0.3523 (15)	−0.2127 (3)	0.0307 (7)	0.071 (2)	0.58 (3)
C5'	0.2903 (15)	−0.1437 (7)	0.0835 (6)	0.077 (2)	0.58 (3)
H5'A	0.2745	−0.1650	0.1337	0.092*	0.58 (3)
C6'	0.2521 (14)	−0.0427 (7)	0.0614 (5)	0.061 (2)	0.58 (3)
H6'A	0.2107	0.0035	0.0968	0.073*	0.58 (3)
F2	0.79946 (16)	0.38529 (10)	0.18219 (8)	0.0850 (4)	
F3	0.46235 (17)	0.66668 (10)	0.24915 (8)	0.0815 (4)	
O1	0.14882 (15)	0.15186 (9)	0.01943 (6)	0.0446 (3)	
O2	0.18983 (15)	0.37207 (9)	0.08958 (6)	0.0447 (3)	
O3	0.27950 (16)	0.45938 (9)	−0.01226 (6)	0.0511 (3)	
O4	0.35555 (19)	0.43640 (13)	0.22503 (7)	0.0758 (5)	
N1	0.4384 (2)	0.46921 (12)	0.10395 (8)	0.0447 (4)	
H1A	0.514 (2)	0.5000 (13)	0.0829 (10)	0.042 (5)*	
C7	0.2094 (2)	0.09129 (15)	−0.03758 (10)	0.0475 (4)	
C8	0.2100 (3)	0.14741 (17)	−0.10359 (11)	0.0615 (5)	
H8	0.2445	0.1244	−0.1509	0.074*	
C9	0.1489 (3)	0.24670 (16)	−0.08727 (10)	0.0597 (5)	
H9	0.1361	0.3017	−0.1216	0.072*	
C10	0.1125 (2)	0.24690 (14)	−0.01267 (9)	0.0448 (4)	
C11	0.0465 (2)	0.32610 (14)	0.03827 (10)	0.0480 (4)	
H11A	−0.0405	0.2956	0.0695	0.058*	
H11B	−0.0121	0.3798	0.0068	0.058*	
C12	0.2968 (2)	0.43425 (12)	0.05510 (9)	0.0406 (4)	
C13	0.4595 (2)	0.47122 (13)	0.18313 (9)	0.0440 (4)	
C14	0.6266 (2)	0.52450 (13)	0.21432 (8)	0.0424 (4)	
C15	0.7915 (3)	0.48058 (15)	0.21366 (10)	0.0542 (5)	
C16	0.9447 (3)	0.5274 (2)	0.24403 (12)	0.0678 (6)	
H16	1.0543	0.4951	0.2427	0.081*	
C17	0.9310 (3)	0.6231 (2)	0.27633 (11)	0.0710 (6)	
H17	1.0332	0.6562	0.2971	0.085*	
C18	0.7704 (3)	0.67107 (17)	0.27869 (11)	0.0642 (6)	
H18	0.7620	0.7361	0.3006	0.077*	
C19	0.6231 (3)	0.62066 (15)	0.24798 (10)	0.0518 (5)	

### Atomic displacement parameters (Å^2^)

**Table:**

	*U*^11^	*U*^22^	*U*^33^	*U*^12^	*U*^13^	*U*^23^
F1	0.092 (4)	0.048 (3)	0.099 (5)	0.015 (2)	0.008 (3)	−0.003 (3)
C1	0.012 (4)	0.049 (4)	0.047 (3)	−0.015 (2)	−0.004 (3)	−0.016 (3)
C2	0.040 (5)	0.064 (5)	0.063 (4)	−0.007 (3)	−0.009 (4)	−0.015 (3)
C3	0.062 (5)	0.084 (6)	0.061 (4)	−0.012 (4)	0.010 (4)	−0.016 (3)
C4	0.045 (4)	0.048 (5)	0.071 (5)	−0.005 (3)	0.010 (3)	0.001 (3)
C5	0.048 (4)	0.036 (4)	0.061 (4)	0.004 (3)	0.006 (3)	0.007 (3)
C6	0.040 (4)	0.032 (3)	0.057 (4)	−0.006 (3)	0.011 (3)	−0.019 (3)
F1'	0.118 (3)	0.068 (3)	0.142 (6)	0.020 (2)	0.013 (5)	−0.022 (3)
C1'	0.024 (4)	0.057 (3)	0.057 (3)	−0.017 (2)	−0.002 (2)	−0.022 (2)
C2'	0.037 (3)	0.069 (4)	0.062 (3)	0.001 (2)	−0.002 (3)	−0.026 (2)
C3'	0.052 (3)	0.054 (4)	0.089 (5)	0.003 (3)	0.004 (3)	−0.035 (3)
C4'	0.051 (3)	0.053 (4)	0.109 (5)	0.004 (3)	0.009 (4)	−0.031 (4)
C5'	0.076 (4)	0.072 (4)	0.084 (4)	−0.001 (3)	0.016 (3)	−0.028 (3)
C6'	0.062 (4)	0.051 (3)	0.069 (4)	−0.007 (3)	−0.001 (3)	−0.016 (3)
F2	0.0776 (9)	0.0746 (9)	0.1041 (10)	0.0198 (7)	0.0145 (7)	−0.0250 (7)
F3	0.0814 (9)	0.0739 (8)	0.0900 (9)	0.0234 (7)	0.0129 (7)	−0.0138 (7)
O1	0.0467 (7)	0.0500 (7)	0.0377 (6)	−0.0049 (5)	0.0076 (5)	−0.0051 (5)
O2	0.0493 (7)	0.0469 (7)	0.0385 (6)	−0.0105 (5)	0.0074 (5)	0.0014 (5)
O3	0.0586 (8)	0.0567 (8)	0.0374 (6)	−0.0106 (6)	0.0007 (5)	0.0091 (6)
O4	0.0671 (9)	0.1219 (13)	0.0399 (7)	−0.0305 (9)	0.0139 (7)	0.0033 (7)
N1	0.0487 (9)	0.0521 (9)	0.0340 (7)	−0.0146 (7)	0.0074 (6)	0.0037 (6)
C7	0.0425 (10)	0.0562 (12)	0.0446 (10)	−0.0107 (8)	0.0087 (8)	−0.0159 (8)
C8	0.0718 (13)	0.0758 (15)	0.0388 (10)	−0.0165 (11)	0.0153 (9)	−0.0127 (10)
C9	0.0743 (13)	0.0650 (13)	0.0402 (10)	−0.0149 (10)	0.0071 (9)	0.0002 (9)
C10	0.0445 (10)	0.0492 (11)	0.0407 (9)	−0.0095 (8)	0.0030 (7)	−0.0008 (8)
C11	0.0415 (10)	0.0544 (11)	0.0484 (10)	−0.0075 (8)	0.0048 (8)	−0.0012 (8)
C12	0.0451 (10)	0.0395 (9)	0.0380 (9)	−0.0020 (7)	0.0073 (7)	0.0009 (7)
C13	0.0470 (10)	0.0512 (10)	0.0348 (8)	0.0002 (8)	0.0098 (7)	0.0020 (8)
C14	0.0466 (10)	0.0522 (11)	0.0289 (8)	0.0009 (8)	0.0065 (7)	0.0004 (7)
C15	0.0585 (12)	0.0591 (12)	0.0456 (10)	0.0072 (9)	0.0077 (9)	−0.0039 (9)
C16	0.0449 (12)	0.0960 (18)	0.0613 (12)	0.0041 (11)	−0.0034 (9)	0.0085 (12)
C17	0.0709 (15)	0.0912 (18)	0.0484 (11)	−0.0217 (13)	−0.0109 (10)	0.0005 (11)
C18	0.0870 (16)	0.0616 (13)	0.0431 (10)	−0.0113 (12)	−0.0012 (10)	−0.0085 (9)
C19	0.0588 (12)	0.0580 (12)	0.0389 (9)	0.0066 (9)	0.0058 (8)	−0.0021 (8)

### Geometric parameters (Å, °)

**Table:**

F1—C4	1.327 (6)	O1—C10	1.372 (2)
C1—C2	1.3900	O2—C12	1.3196 (18)
C1—C6	1.3900	O2—C11	1.4710 (19)
C1—C7	1.452 (4)	O3—C12	1.2091 (18)
C2—C3	1.3900	O4—C13	1.2008 (19)
C2—H2A	0.9300	N1—C13	1.369 (2)
C3—C4	1.3900	N1—C12	1.386 (2)
C3—H3A	0.9300	N1—H1A	0.810 (17)
C4—C5	1.3900	C7—C8	1.357 (3)
C5—C6	1.3900	C8—C9	1.406 (3)
C5—H5A	0.9300	C8—H8	0.9300
C6—H6A	0.9300	C9—C10	1.343 (2)
F1'—C4'	1.347 (6)	C9—H9	0.9300
C1'—C2'	1.3900	C10—C11	1.469 (2)
C1'—C6'	1.3900	C11—H11A	0.9700
C1'—C7	1.466 (4)	C11—H11B	0.9700
C2'—C3'	1.3900	C13—C14	1.502 (2)
C2'—H2'A	0.9300	C14—C15	1.372 (2)
C3'—C4'	1.3900	C14—C19	1.380 (2)
C3'—H3'A	0.9300	C15—C16	1.375 (3)
C4'—C5'	1.3900	C16—C17	1.371 (3)
C5'—C6'	1.3900	C16—H16	0.9300
C5'—H5'A	0.9300	C17—C18	1.369 (3)
C6'—H6'A	0.9300	C17—H17	0.9300
F2—C15	1.356 (2)	C18—C19	1.362 (3)
F3—C19	1.356 (2)	C18—H18	0.9300
O1—C7	1.3705 (19)		
			
C2—C1—C6	120.0	O1—C7—C1	117.9 (4)
C2—C1—C7	115.9 (5)	C8—C7—C1'	134.4 (3)
C6—C1—C7	122.9 (5)	O1—C7—C1'	116.2 (3)
C1—C2—C3	120.0	C1—C7—C1'	11.5 (9)
C1—C2—H2A	120.0	C7—C8—C9	107.42 (16)
C3—C2—H2A	120.0	C7—C8—H8	126.3
C4—C3—C2	120.0	C9—C8—H8	126.3
C4—C3—H3A	120.0	C10—C9—C8	106.95 (18)
C2—C3—H3A	120.0	C10—C9—H9	126.5
F1—C4—C3	122.2 (6)	C8—C9—H9	126.5
F1—C4—C5	117.4 (6)	C9—C10—O1	109.79 (16)
C3—C4—C5	120.0	C9—C10—C11	133.30 (18)
C6—C5—C4	120.0	O1—C10—C11	116.91 (14)
C6—C5—H5A	120.0	C10—C11—O2	112.23 (14)
C4—C5—H5A	120.0	C10—C11—H11A	109.2
C5—C6—C1	120.0	O2—C11—H11A	109.2
C5—C6—H6A	120.0	C10—C11—H11B	109.2
C1—C6—H6A	120.0	O2—C11—H11B	109.2
C2'—C1'—C6'	120.0	H11A—C11—H11B	107.9
C2'—C1'—C7	121.5 (4)	O3—C12—O2	125.48 (15)
C6'—C1'—C7	117.9 (5)	O3—C12—N1	121.25 (15)
C3'—C2'—C1'	120.0	O2—C12—N1	113.27 (14)
C3'—C2'—H2'A	120.0	O4—C13—N1	124.77 (17)
C1'—C2'—H2'A	120.0	O4—C13—C14	121.89 (15)
C2'—C3'—C4'	120.0	N1—C13—C14	113.32 (14)
C2'—C3'—H3'A	120.0	C15—C14—C19	115.38 (17)
C4'—C3'—H3'A	120.0	C15—C14—C13	122.94 (16)
F1'—C4'—C5'	121.2 (5)	C19—C14—C13	121.66 (15)
F1'—C4'—C3'	118.6 (5)	F2—C15—C14	116.95 (17)
C5'—C4'—C3'	120.0	F2—C15—C16	119.58 (18)
C6'—C5'—C4'	120.0	C14—C15—C16	123.46 (19)
C6'—C5'—H5'A	120.0	C17—C16—C15	117.9 (2)
C4'—C5'—H5'A	120.0	C17—C16—H16	121.1
C5'—C6'—C1'	120.0	C15—C16—H16	121.1
C5'—C6'—H6'A	120.0	C18—C17—C16	121.5 (2)
C1'—C6'—H6'A	120.0	C18—C17—H17	119.3
C7—O1—C10	106.94 (13)	C16—C17—H17	119.3
C12—O2—C11	115.00 (12)	C19—C18—C17	117.9 (2)
C13—N1—C12	129.79 (15)	C19—C18—H18	121.0
C13—N1—H1A	114.4 (12)	C17—C18—H18	121.0
C12—N1—H1A	115.2 (12)	F3—C19—C18	119.16 (18)
C8—C7—O1	108.89 (17)	F3—C19—C14	116.96 (17)
C8—C7—C1	132.8 (4)	C18—C19—C14	123.87 (18)
			
C6—C1—C2—C3	0.0	O1—C7—C8—C9	0.4 (2)
C7—C1—C2—C3	167.9 (11)	C1—C7—C8—C9	172.9 (8)
C1—C2—C3—C4	0.0	C1'—C7—C8—C9	−171.4 (8)
C2—C3—C4—F1	172.2 (14)	C7—C8—C9—C10	−0.4 (2)
C2—C3—C4—C5	0.0	C8—C9—C10—O1	0.3 (2)
F1—C4—C5—C6	−172.6 (13)	C8—C9—C10—C11	179.54 (18)
C3—C4—C5—C6	0.0	C7—O1—C10—C9	−0.07 (18)
C4—C5—C6—C1	0.0	C7—O1—C10—C11	−179.44 (14)
C2—C1—C6—C5	0.0	C9—C10—C11—O2	−100.7 (2)
C7—C1—C6—C5	−167.1 (12)	O1—C10—C11—O2	78.50 (18)
C6'—C1'—C2'—C3'	0.0	C12—O2—C11—C10	72.38 (18)
C7—C1'—C2'—C3'	−170.9 (11)	C11—O2—C12—O3	4.2 (2)
C1'—C2'—C3'—C4'	0.0	C11—O2—C12—N1	−174.94 (13)
C2'—C3'—C4'—F1'	−175.4 (12)	C13—N1—C12—O3	162.21 (17)
C2'—C3'—C4'—C5'	0.0	C13—N1—C12—O2	−18.6 (3)
F1'—C4'—C5'—C6'	175.3 (12)	C12—N1—C13—O4	3.9 (3)
C3'—C4'—C5'—C6'	0.0	C12—N1—C13—C14	−174.66 (16)
C4'—C5'—C6'—C1'	0.0	O4—C13—C14—C15	106.4 (2)
C2'—C1'—C6'—C5'	0.0	N1—C13—C14—C15	−75.0 (2)
C7—C1'—C6'—C5'	171.2 (10)	O4—C13—C14—C19	−71.6 (2)
C10—O1—C7—C8	−0.21 (18)	N1—C13—C14—C19	107.02 (18)
C10—O1—C7—C1	−174.0 (7)	C19—C14—C15—F2	178.90 (15)
C10—O1—C7—C1'	173.2 (6)	C13—C14—C15—F2	0.8 (2)
C2—C1—C7—C8	10.4 (11)	C19—C14—C15—C16	0.0 (3)
C6—C1—C7—C8	177.9 (5)	C13—C14—C15—C16	−178.07 (17)
C2—C1—C7—O1	−177.7 (5)	F2—C15—C16—C17	−179.09 (18)
C6—C1—C7—O1	−10.1 (11)	C14—C15—C16—C17	−0.2 (3)
C2—C1—C7—C1'	−93 (3)	C15—C16—C17—C18	0.2 (3)
C6—C1—C7—C1'	75 (3)	C16—C17—C18—C19	0.1 (3)
C2'—C1'—C7—C8	−9.8 (11)	C17—C18—C19—F3	−179.62 (17)
C6'—C1'—C7—C8	179.1 (4)	C17—C18—C19—C14	−0.4 (3)
C2'—C1'—C7—O1	178.8 (5)	C15—C14—C19—F3	179.55 (15)
C6'—C1'—C7—O1	7.8 (8)	C13—C14—C19—F3	−2.3 (2)
C2'—C1'—C7—C1	78 (3)	C15—C14—C19—C18	0.3 (3)
C6'—C1'—C7—C1	−93 (3)	C13—C14—C19—C18	178.41 (16)

### Hydrogen-bond geometry (Å, °)

**Table:**

*D*—H···*A*	*D*—H	H···*A*	*D*···*A*	*D*—H···*A*
N1—H1A···O3^i^	0.810 (17)	2.126 (18)	2.9129 (19)	164.0 (17)

Symmetry codes: (i) −*x*+1, −*y*+1, −*z*.

## References

[bb1] Bruker (1999). *SAINT* and *SMART* Bruker AXS Inc., Madison, Wisconsin, USA.

[bb2] Grosscurt, A. C. & Tipker, J. (1980). *Pestic. Biochem. Physiol.* **13**, 249–254.

[bb3] Grugier, J., Xie, J., Duarte, I. & Valery, J. M. (2000). *J. Org. Chem.* **65**, 979–984. 10.1021/jo991206p 10814043

[bb4] Li, Y., Cui, Z. N., Hu, J., Ling, Y. & Yang, X. L. (2007). *Prog. Chem. ***19**, 535–543.

[bb5] Sheldrick, G. M. (1996). *SADABS* University of Göttingen, Germany.

[bb6] Sheldrick, G. M. (2008). *Acta Cryst.* A**64**, 112–122. 10.1107/S0108767307043930 18156677

[bb7] Yang, X. L., Ling, Y., Wang, D. Q. & Chen, F. H. (2002). *Chin. J. Synth. Chem.* **10**, 510–512.

[bb8] Yang, X. L., Wang, D. Q., Chen, F. H., Ling, Y. & Zhang, Z. N. (1998). *Pestic. Sci.* **52**, 282–286.

[bb9] Yang, X. L., Wang, D. Q., Chen, F. H., Ling, Y., Zhang, Z. N. & Shang, Z. Z. (1997). *Chem. J. Chin. Univ.* **18**, 395–398.

